# CRISPR/Cas9 in colorectal cancer: Revolutionizing precision oncology through genome editing and targeted therapeutics

**DOI:** 10.22038/ijbms.2025.87531.18902

**Published:** 2025

**Authors:** Bahjat Alhasso, Abdulkareem Shareef, Lalji Baldaniya, Rami Oweis, Renuka Jyothi, Udaybir Singh, Samir Sahoo, Ashish Singh Chauhan, Hayder Naji Sameer, Ahmed Yaseen, Zainab H. Athab, Mohaned Adil

**Affiliations:** 1 College of Pharmacy, Alnoor University, Nineveh, Iraq; 2 Ahl al bayt University, Karbala, Iraq; 3 Marwadi University Research Center, Department of Pharmaceutical Sciences, Faculty of Health Sciences, Marwadi University, Rajkot, Gujarat, India; 4 Department of Pharmacy, Modern College of Business and Science, Muscat, Oman; 5 Department of Biotechnology and Genetics, School of Sciences, JAIN (Deemed to be University), Bangalore, Karnataka, India; 6 Centre for Research Impact & Outcome, Chitkara University Institute of Engineering and Technology, Chitkara University, Rajpura, 140401, Punjab, India; 7 Department of General Medicine, IMS and SUM Hospital, Siksha ‘O’ Anusandhan (Deemed to be University), Bhubaneswar, Odisha-751003, India; 8 Uttaranchal Institute of Pharmaceutical Sciences, Division of Research and Innovation, Uttaranchal University, Dehradun, Uttarakhand, India; 9 Collage of Pharmacy, National University of Science and Technology, Dhi Qar, 64001, Iraq; 10 Gilgamesh Ahliya University, Baghdad, Iraq; 11 Department of Pharmacy, Al-Zahrawi University College, Karbala, Iraq; 12 Pharmacy college, Al-Farahidi University, Baghdad, Iraq

**Keywords:** CRISPR/Cas9, Colorectal Cancer (CRC) Gene Therapy, Genome Editing, Oncogenic Pathways, Tumor Suppression

## Abstract

Colorectal cancer (CRC) remains a significant global health challenge, necessitating advanced molecular therapies to improve outcomes. The CRISPR/Cas9 genome-editing platform has emerged as a transformative tool in CRC research, enabling precise genomic modifications to suppress tumor progression, enhance chemosensitivity, and modulate oncogenic pathways. This review highlights CRISPR/Cas9 applications in CRC models, including MC38 murine and CaCO-2 cell lines, where targeted gene edits demonstrate tumor-suppressive effects. For instance, Par3L protein knockout via CRISPR/Cas9 inhibits proliferation, induces apoptosis, and sensitizes cells to chemotherapy by regulating AMPK signaling. Additionally, AAV-mediated CRISPR editing shows promise in HPV16-driven CRC models. Despite its potential, clinical translation faces challenges such as off-target effects, immunogenicity, and delivery limitations. Advances in engineered CRISPR variants (e.g., xCas9, HypaCas9) and innovative delivery systems are refining specificity and efficacy. CRISPR/Cas9 also accelerates biomarker discovery, paving the way for precision oncology. Overcoming current barriers could revolutionize CRC therapeutics, offering personalized treatment paradigms.

## Introduction

The third most common cancer and the fourth leading cause of cancer-related death globally, colorectal cancer (CRC), is on the rise, particularly in Western nations. The lifetime risk is estimated to be 4-5% and is influenced by factors such as age, lifestyle, and medical history. Dysbiosis, or imbalances in the gut microbiota, is linked to CRC by promoting chronic inflammation and tumor growth, with species such as *Escherichia coli, Bacteroides fragilis, and Fusobacterium* spp (1).

In the late 1980s, gene therapy became a novel method of replacing or repairing damaged genes. Although it may be used on both plants and animals, its primary focus is treating humans. Scientists and the pharmaceutical industry are very interested in gene therapy because it can potentially treat or perhaps cure several hereditary illnesses by focusing on the underlying genetic causes of disease (2, 3). The CRISPR/Cas9 system has been developed into a potent gene editing tool due to extensive study (2, 4-13).

Although extensive genetic research has shed light on the links between genetic variants and illnesses for many years, it is still difficult to pinpoint the exact processes by which these differences materialize as specific ailments. Direct changes to the genome are necessary to correct mutations to treat such conditions, but making accurate genetic changes within the large and complex human genome has always been a major scientific challenge. Each cell’s nucleus has almost one meter of DNA, containing thousands of protein-coding and noncoding genes that comprise the human genome (14).

With a focus on precise genome modifications at specific target locations, genetic engineering has advanced significantly in recent years. Thanks to these developments that have transformed the industry, genetic engineering is emerging as a vital tool in preclinical research and may someday provide patients with currently incurable illnesses with new therapeutic choices. The advent of the CRISPR/Cas9 system (Clustered Regularly Interspaced Short Palindromic Repeat/CRISPR-associated protein 9) was one of the most important advancements in genome editing. 

This innovative method allows precise and efficient genome editing or control, providing unparalleled adaptability. Enhancing our understanding of how genes function in the human genome and paving the way for novel therapeutic strategies depend on CRISPR/Cas9 (15). 

Through bioinformatics-driven screening, CRISPR/Cas9-based screening library technology has emerged as a potent instrument in cancer research. Various experimental models, such as 2D cell lines, 3D organoids, and animal models, are commonly used in genome-wide investigations (16, 17). The advancement of CRISPR/Cas9 technology has been a major contributor to the increased effectiveness of CAR-T cell therapy. Dongrui *et al*.’s 2021 study employed genome-wide screening in CAR-T and glioma stem cells (GSCs) to identify genetic changes that may enhance therapeutic outcomes. Their findings demonstrated that TLE4 and IKZF2 deletion significantly enhanced CAR-T cells’ anti-tumor activity, suggesting that CRISPR/Cas9 may enhance cancer immunotherapy (10, 18).

The accumulation of genetic and epigenetic changes that impact important pathways like Wnt/β-catenin, MAPK, and PI3K, along with frequent mutations in genes like APC, KRAS, and TP53, causes CRC. Tumor start, progression, and therapeutic resistance are all influenced by these alterations. Many patients with advanced or metastatic CRC have few treatment choices and low survival rates, even with advancements in surgery, chemotherapy, and targeted medications. Current treatments frequently fail to address the underlying genetic causes and tumor heterogeneity. A unique approach to directly target and fix mutations linked to CRC is provided by the highly precise and versatile genome-editing technology CRISPR/Cas9. One intriguing avenue for overcoming present therapeutic constraints is its capacity to model illness and create customized gene-based therapies (19, 20).

This study intends to thoroughly examine the uses of CRISPR/Cas9 in CRC research and treatment, emphasizing its potential in immunotherapy, tumor suppression, and targeted gene changes. In particular, Scientists have investigated how well CRISPR/Cas9 functions across various CRC models, its impact on therapy response, and its role in modulating key carcinogenic pathways. Along with discussing the current challenges with CRISPR-based genome editing, including off-target effects, immunogenic responses, and transport constraints, we also look at recent advancements in customized CRISPR variants and nanoparticle-based delivery systems. Using the most current developments in the field, this study aims to demonstrate the revolutionary potential of CRISPR/Cas9 technology in treating CRC and its promise for precision oncology in the future.

### CRISPR-Cas9: Mechanism and application in cancer research

*How CRISPR-Cas9 works: DNA targeting, cutting, and repair*

Target sequence identification, precise endonucleolytic breaking, and subsequent DNA repair through cellular mechanisms are the three essential phases in the CRISPR/Cas9 genome-editing process (21, 22). By attaching itself to the complementary 5′ crRNA region, the designated single-guide RNA (sgRNA) acts as a molecular guide, directing the Cas9 enzyme to the precise genomic sequence of interest. In the absence of sgRNA, the Cas9 protein is inactive. Proper placement of the Cas9 nuclease results in a precise double-strand break (DSB) three base pairs upstream of the protospacer adjacent motif (PAM)(22).

Directly downstream of the cleavage site is a conserved DNA sequence called PAM, which typically has two to five base pairs. The type of bacteria determines its exact length and composition. The PAM sequence, which is frequently encoded as 5′-NGG-3′ (where “N” stands for any nucleotide), is recognized and bound by the Cas9 protein, the most widely used nuclease in genome-editing applications. When Cas9 locates a target sequence next to a suitable PAM and initiates local DNA unwinding, an RNA-DNA hybrid is produced (22). 

**Figure 1:**
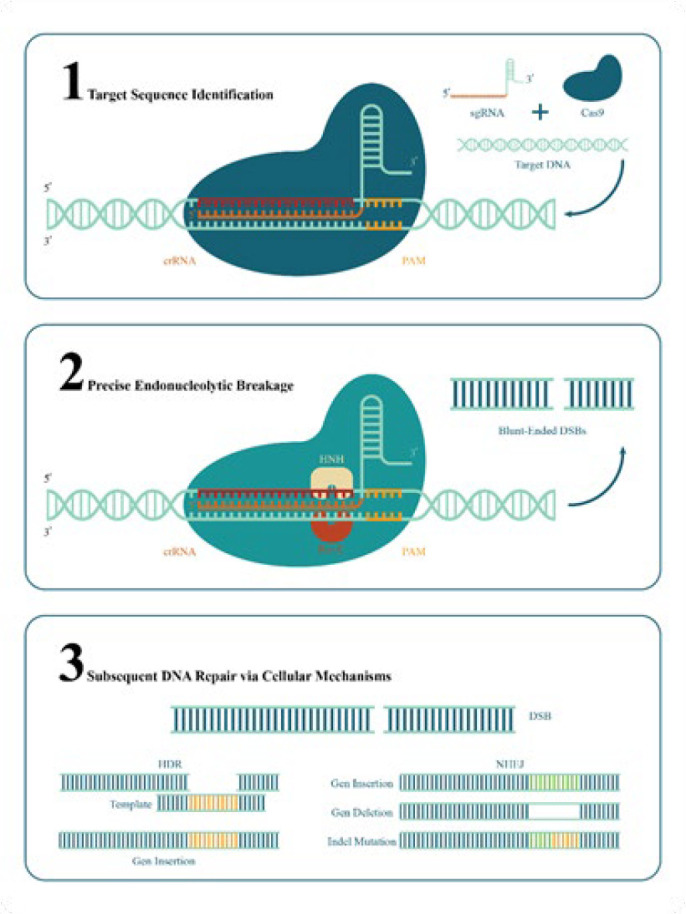
CRISPR/Cas9 genome editing mechanism

The exact chemical mechanism underlying this DNA melting process is unknown. Cas9 is activated to break DNA when the target has been successfully identified. The enzyme’s HNH domain cuts the complementary DNA strand to the guide RNA after the RuvC domain cleaves the non-complementary DNA strand, resulting in DSBs that are mostly blunt-ended. These breaks are subsequently repaired by the host cell’s regular DNA repair mechanisms (Figure 1)(23,24).

*Applications in cancer research: Gene knockout, correction, and screening*

In particular, CRISPR-based gene editing provides valuable insights into altered glucose, lipid, and amino acid metabolism, mitochondrial function, and energy production, shedding light on the complex processes of metabolic reprogramming in cancer cells. This technology, especially gene knockout, holds great promise for clinically targeting tumor-related genes. It enables precise alterations of both tumor suppressor genes and oncogenes, supports the identification of new therapeutic targets related to cancer stem cells (CSCs), and deepens our understanding of metabolic disruptions in cancer (27, 28).

Subsequent investigation revealed that PUM1 is required to regulate DDX5, positively improving cell survival. According to these results, PUM1 and DDX5 expression reduction may reduce tumor cell survival, which makes them promising therapeutic targets to make colon cancer cells more susceptible to trastuzumab therapy (Table 1)(27, 29).

WHSC1 has been demonstrated to be an oncogenic factor and transcriptional target of HMGA2, and both HMGA2 and WHSC1 regulate the proliferation of cancer cells. This connection increases the likelihood of metastasis and promotes the growth of cancer cells. Targeted CRISPR-mediated deletion of WHSC1 in colon cancer cells has been shown to inhibit the growth of tumor cells, enhance sensitivity to treatment medications, and significantly reduce the tumor cells’ capacity to spread (27, 30).

The TFAP2A gene encodes activated protein 2α (AP-2α), a tumor suppressor implicated in the transcriptional control of colon cancer. Beck *et al*. used shRNA and CRISPR/Cas9 to suppress TFAP2A expression in HCT116 and other colon cancer cell lines. Tumor cells demonstrated resistance to the PI3K inhibitor buparlisib (BKM120) when AP-2α expression was decreased, suggesting that elevated AP-2α levels may increase tumor cells’ susceptibility to buparlisib/BKM120 therapy (26, 27).

*Specific genes implicated in CRC targeted by CRISPR*

It is well-accepted that CRC is a diverse disease caused by various genetic alterations and the activation of several oncogenic pathways (30). The most common are KRAS mutations, which occur in around 40% of cases, and BRAF mutations, which occur in about 10% of cases (31, 32).

Patients with KRAS or BRAF mutations frequently have worse survival rates and have reduced responsiveness to a range of treatment modalities, including radiation, chemotherapy, targeted therapies, and immunotherapy, in comparison to those with wild-type genotypes (31-40).

**Figure 2:**
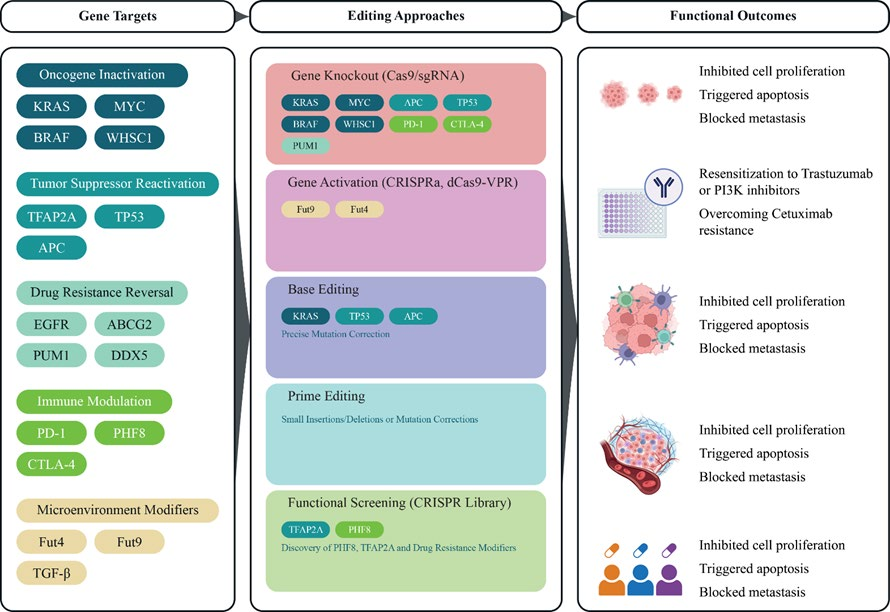
CRISPR/Cas9-based gene editing strategies in colorectal cancer

Researchers used a CRISPR-Cas9 gene knockout library to identify the histone lysine demethylase PHF8 as a promising immunotherapeutic target. By transcriptionally up-regulating the expression of KRAS, BRAF, and c-Myc, we demonstrate that PHF8 predominantly has an oncogenic effect in KRAS- or BRAF-mutant CRC cells but not in wild-type cells. We do this by effectively suppressing the growth of tumors by reducing the expression of PD-L1. According to these results, cells with BRAF or KRAS mutations may benefit from PHF8 as a therapeutic target (Table 2)(Figure 2)(31).

### Promises of CRISPR-Cas9 in CRC therapy

*Lung-directed CRISPR gene editing highlights therapeutic potential for CRC lung metastases while underscoring the limits of NSCLC-based models*

Includes TP53, KRAS, and APC gene alterations connected to CRC. One of the leading causes of non-small cell lung cancer (NSCLC) in humans is a combination of activating mutations in important parts of the MAPK pathway, particularly KRAS changes and TP53 deletion. We compared the Rosa26Sor-CAG-Cas9-IRES-eGFP mice (C57BL6/J background) infected with an AAV expressing sgRNA to target Trp53 and induce a KRasG12 mutation with the standard Trp53fl/fl:KRaslsl–G12D/wt mouse model (C57BL6/J background, KPGEMM) infected with an AAV carrying Cre recombinase to evaluate the potential of CRISPR/Cas9 gene editing in simulating NSCLC *in vivo*. Prior research has demonstrated that *in vivo* CRISPR-mediated genome editing provides few off-target changes and great precision (50-52).

Twelve weeks following the intratracheal administration of virus-containing solutions, both KPCRISPR and KPGEMM mice developed tumors; tracheal and oral examination of the infected mice showed no signs of off-target tumor formation; and there were no appreciable differences between KPGEMM and KPCRISPR in terms of tumor cell proliferation, as indicated by the percentage of proliferating cell nuclear antigen (PCNA)-positive cells within the tumors, or tumor burden, as assessed by hematoxylin and eosin (HE) staining. The AAV-DJ variant, which was produced by capsid shuffling many AAV serotypes, had the highest infection effectiveness in CRC models among the evaluated modified adeno-associated virus (AAV) vectors (53). By combining tropisms from AAV serotypes 2, 8, and 9, this hybrid capsid circumvents pre-existing immunity and allows for wider tissue targeting (52).

Then, we looked at any differences in tumor grade between KPCRISPR and KPGEMM. According to the established categorization system for mouse NSCLC, both models had comparable distributions at every stage, from stage I atypical alveolar hyperplasia (AAH) to stage IV (52, 54, 55).

The genetic targeting approach did not affect overall animal survival over the 12-week study period following intratracheal injection. Sanger sequencing of the targeted genomic areas verified the genetic changes caused by CRISPR gene editing (Table 3). The success of KRas targeting was shown by the detection of the KRasG12D HDR template integration. Sanger sequencing revealed that CRISPR editing changed or eliminated the length of the Trp53 gene. The KRasG12D mutation activated the MAPK pathway, and both KPGEMM and KPCRISPR tumors exhibited elevated phosphorylation levels of MAPK1/3 (p-Erk1/2) compared to the surrounding non-tumor tissue. Similar expression patterns were observed in both groups when analyzing lung-specific markers, such as the tracheal club cell marker Scgb1a1 (CC10), the alveolar type II marker surfactant protein C (SftpC), and the adenocarcinoma marker thyroid transcription factor-1 (TTF1). However, a key difference emerged: KPGEMM tumors expressed the basal stem and squamous cell carcinoma marker Krt5, while KPCRISPR tumors did not. Notably, Krt5 was positive in KPGEMM tumors, while Sox2, a marker linked to squamous cell carcinoma, was absent in all samples. Only the basal cells of the trachea showed positive staining, indicating that tumor cells were co-expressed rather than suggesting squamous growth had occurred. Lastly, when the two models were examined for metastases, neither revealed any significant abnormalities or metastases in distant organs such as the liver, pancreas, or intestine (52).

Theoretically, introducing changes into the AAV capsid that inhibit NAb binding is the most attractive way to get around the problem of pre-existing NAbs. However, the high degree of cross-reactivity between AAV serotypes (56, 57) demonstrates how difficult it is to accomplish this. However, it has been shown that adding point mutations to the AAV2 capsid might lessen these mutant viruses’ susceptibility to neutralization (58). However, a significant portion of all neutralizing antibodies against the virus are targeted at the receptor binding domain or domains, which is the case for most viruses and most likely AAV. Mutations in the receptor binding region(s) are, therefore, likely to impact viral tropism and/or transduction efficiency, but they may also be the most effective way to improve neutralization.

Only in patients with low NAb titers has traditional plasmapheresis, which eliminates all immunoglobulins, shown some promise in reducing the majority of NAbs from patient sera (59). Furthermore, eliminating all immunoglobulins has disadvantages of its own. As of late, Bertin *et al*. (60) and Orlowski *et al*. (61) have shown that by incubating IVIG or human sera with beads with AAV particles covalently attached, neutralizing antibodies/factors may be eliminated *in vitro*. Furthermore, we were able to show that using these beads to perform hemapheresis may completely restore liver transduction in animals with NAb titers that exhibit none to very little transduction in the absence of hemapheresis (61). The recovery of cardiac and, particularly, skeletal muscle transduction was less severe, most likely due to NAbs returning to the circulation from the extracellular fluid. Unfortunately, we could not do numerous rounds of hemapheresis over several days due to technical constraints (61). Humans can easily undergo numerous rounds of hemapheresis over a few days (59). Therefore, humans could quickly surpass the rebound constraint in rats. Future studies using large animal models, especially non-human primates, may demonstrate the effectiveness of this strategy.

The streptococcal cysteine protease imlifidase (IdeS), which can cleave IgG into F (ab’) 2 fragments and Fc, was recently exploited by Mingozzi and associates (62, 63). After a 24-hr incubation period, total IgG and anti-AAV8 IgG were completely digested following IVIG treatment with IdeS. Furthermore, Leborgne *et al*. eliminated the expression of either human FIX (hFIX) or secreted Gaussia luciferase when they passively vaccinated mice with human IVIG and then injected them with AAV8 expressing either hFIX or secreted Gaussia luciferase a day later. However, if the mice were given human IVIG on day 0 and IdeS 30 min later, and then AAV8 encoding secreted Gaussia luciferase or hFIX 1 day later, the blood levels of the luciferase or hFIX expression were identical to those of naïve mice (63). Remarkably, IdeS therapy permitted vector re-administration with the same AAV variant (AAV-LK03) in addition to the transduction of NHPs with pre-existing neutralizing antibodies (63).

Asokan and associates used IdeZ, a homolog of IdeS generated by an alternative streptococcal strain. This study might also show that transduction of mice passively inoculated with IVIG is possible when IdeZ is administered. Additionally, they found that an NHP with pre-existing neutralizing antibodies that had previously had an IdeZ injection was successfully transduced with HPV (64).

### CRISPR-Based Immunotherapy

*Enhancing T-cell response against CRC tumors*

The presence of CD3+ and CD8+ cytotoxic T cells within the tumor core or at its edges, known as the immunoscore, is one of the most reliable prognostic indicators for both recurrence and overall survival in CRC. The tumor microenvironment (TME) is a critical component in both cancer progression and tumor eradication. The complicated interactions between cancer cells and different non-tumor cells, such as innate and adaptive immune cells, make CRC more than merely a hereditary illness (76, 77).

A prior study by the International Society for Immunotherapy of Cancer (SITC) involving 1,885 patients with stage I–II colon cancer found that the presence of T cells within the tumor core or at its edges is associated with a longer recurrence-free period and improved overall survival, even in smaller tumors (78).

Even in patients without apparent metastases, the presence of CD3+ and CD8+ cells was found to be a good prognostic predictor. In a different study, the same team showed that 763 patients with stage III CRC with a high immunoscore had a greater overall survival rate and a decreased probability of recurrence, highlighting the crucial role T cells play in CRC prognosis (76, 77).

CD8+ and CD4+ T cells are the predominant immune cell types involved in CRC. Most T cells express a T cell receptor (TCR) composed of alpha and beta chains. During their development in the thymus, autoreactive T cells are eliminated through apoptosis as part of a selection process that trains them to distinguish self from non-self. Once mature, naïve T cells migrate to secondary lymphoid organs, where they are activated by antigen-presenting cells (APCs). In tumor-draining lymph nodes, activated APCs, particularly dendritic cells, present tumor antigens via MHC class I molecules to CD8+ T cells and via MHC class II molecules to CD4+ T cells. This antigen-specific interaction triggers the differentiation of cytotoxic CD8+ and helper CD4+ T cells into effector cells, initiating a strong and targeted immune response (77, 79).

The role of conventional CD4+ T cells, which express a T cell receptor composed of alpha and beta chains, is complex in the context of CRC. This complexity arises from the presence of multiple T helper cell subsets, each carrying out unique functions. While some subsets contribute to anti-tumor immunity, others may support tumor progression. Moreover, CD4+ T cells exhibit considerable plasticity, allowing them to quickly adapt their behavior in response to environmental cues (77, 80, 81). 

For example, Th17 cells can change and adopt traits from other T helper cell types (77, 82). CD4+ T cells differentiate into effector and memory T cells upon APC activation (77, 83).

**Figure 3:**
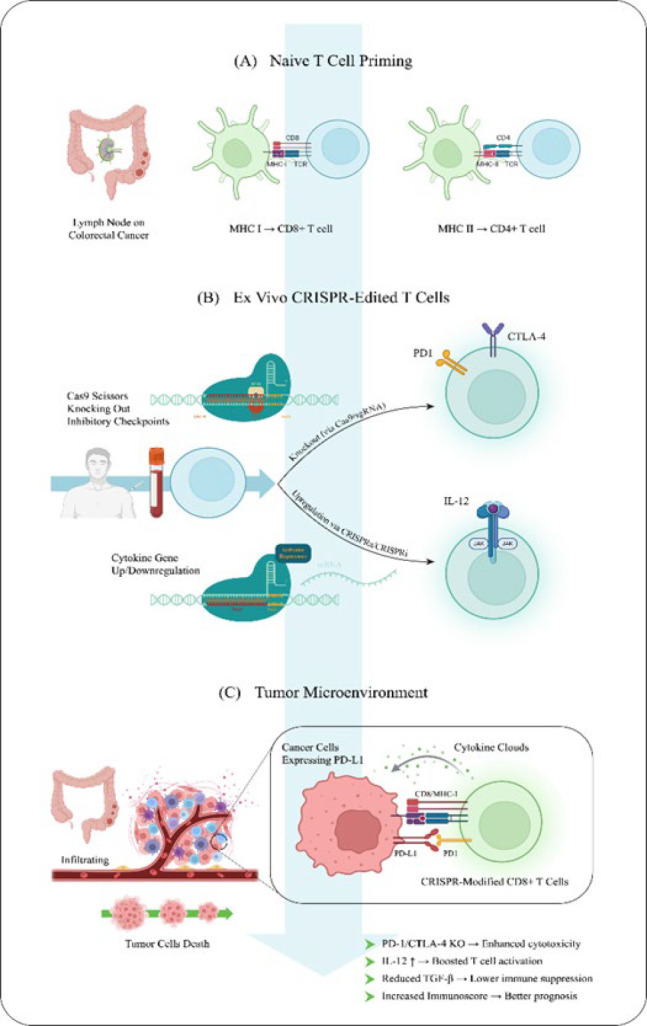
CRISPR/Cas9-enhanced immunotherapy strategies for colorectal cancer (CRC). This figure illustrates the immunological basis and genetic engineering potential of CRISPR/Cas9 in enhancing T-cell-mediated responses against colorectal tumors

Three essential signals are required to activate and polarize a naïve CD4+ T cell: (I) the T cell receptor’s interaction with the APC’s MHC class II complex; (II) a co-stimulatory signal that is not dependent on the antigen, such as the interaction between the T cell’s CD28 molecule and the APC’s CD80/CD86 molecules; and (III) environmental cytokines, which are produced mainly by the APCs (Figure 3A)(77).

CRC tumors are known for having a “cold” or immunosuppressive microenvironment that limits the effectiveness of the immune system’s response to the cancer. For example, CRC tumors often express PD-L1 on their surfaces, which binds to PD-1 on T-cells, inhibiting the immune system’s ability to kill the tumor cells.

After CRISPR edits, T-cells reintroduced to the body can overcome these immunosuppressive signals. For example, by knocking out PD-1 in T-cells, even if the tumor still expresses PD-L1, the T-cells can continue to attack the tumor. Additionally, by modulating cytokine levels, such as up-regulating IL-12, CRISPR-edited T-cells can promote a more active immune response. By reducing TGF-β, the edited T-cells can avoid the immune suppression typical of the CRC tumor environment. Impact on Immunoscore and Patient Prognosis: The changes in T-cell behavior contribute to the “Immunoscore,” which is a clinical measure of immune activity within tumors. A high Immunoscore is correlated with better outcomes in CRC patients, such as improved survival and reduced recurrence. The CRISPR-edited T-cells, with enhanced anti-tumor activity, can lead to an improved Immunoscore and better clinical outcomes for patients with CRC (41, 76, 77, 79).

CD4+ T cells develop into diverse subsets in CRC, including follicular helper cells (Tfh cells), induced or natural regulatory T cells (iTregs and nTregs), Th1, Th2, Th17, and Th22. Specific transcription factors, including T-BET for Th1 cells and GATA-3 for Th2 cells, as well as unique cytokines, such as IL-4 from Th2 cells or IL-17 from Th17 cells, define these subsets apart. Furthermore, specific chemokine receptors and signal transducer and activator of transcription (STAT) proteins help define the roles of each subgroup (77, 80, 84). 

As discussed, T helper cells, especially Th17 cells, are very malleable. Th17 cells generated *in vitro* can mature into Th1 cells that release IFN-γ in conditions like colitis following adoptive transfer (77, 85, 86). Interleukin 22 (IL-22) has been linked to chemotherapy resistance in CRC patients (87) and promotes tumor formation in CRC mouse models (77, 88, 89).

In lab settings, the cytokine transforming growth factor-beta (TGF-β) prevents Th22 cell development, whereas, *in vivo* organisms, it increases Th17 cells’ IL-22 production (90). As a result, Th17 cells can differentiate into Th22 cells. Tregs may also shift phenotypically; by down-regulating FOXP3 expression, they can become ex-Tregs resembling Th1 or Th17 cells (77, 91, 92).

Th1 cells and the cytokines they generate are linked to a better prognosis in CRC. Th1 cells contribute to this by inhibiting the development of cancer cells, partly by lowering angiogenesis, attracting cytotoxic CD8+ T cells, and producing senescence, which promotes the death of cancer cells (77, 93-95). 

While Th1 cells stimulate CD8+ T cell activation, which aids in the anti-tumoral response (66), they also secrete IFN-γ, which increases the expression of checkpoint inhibitors like PD-1 on CD8+ T cells (96, 97).

There is ongoing discussion on Th2 cell involvement in CRC. Th2 cytokines, or pro-inflammatory molecules, such as IL-4, IL-5, and IL-13, aid in sustaining inflammation, which can subsequently encourage the emergence of inflammation-related malignancy (98, 99)(Figure 3). However, Th2 cytokines may attract eosinophils, which have anti-tumoral properties and might help slow cancer progression by promoting changes in the tumor’s vascular structure (Table 4)(77, 100).

Despite encouraging *in vitro* evidence, CAR-T trials in CRC show glaring translational limitations. Dongrui *et al*., for instance, demonstrated 90% cytotoxicity in cell lines (101). However, their xenograft models failed to account for the immunosuppressive CRC tumor microenvironment (TME), which contains PD-L1+ myeloid cells and TGF-β (2.1-4.8 ng/ml)(102). Since just two Phase I studies focus on CRC and fifteen on leukemia, this neglect is clinically relevant and reflects unsolved issues in target validation. Additionally, there are contradictory findings on EpCAM-targeting CAR-Ts: one study observed significant on-target toxicity in normal intestinal epithelia (103), while another study reported regression in peritoneal metastases (104). These differences highlight the necessity for subtype-specific designs considering CMS categorization (105).

### Delivery challenges

*Efficiency of in vivo and ex vivo delivery methods*

Gene delivery methods for *in vivo* applications are generally divided into two main categories: synthetic non-viral vectors and viral vector systems. These approaches can be used either locally or systemically. Although viral vectors are highly efficient at delivering genetic material into target cells, their clinical use is often restricted due to potential cytotoxicity and immune system activation (110-114). Incorporating the CRISPR/Cas9 system into viral vectors is another major challenge. One noteworthy drawback of the widely used AAV vector is its small cargo capacity; it can only hold a maximum of 4.7 kilobase pairs (kbp)(110, 115). Even though SpCas9 and sgRNA may be co-delivered in a single vector, there is little room for donor repair templates and crucial regulatory components due to the limited packing capacity (110, 116, 117).

This constraint can be circumvented by using a shorter version of SpCas9 to reduce its genomic footprint (118, 119). Another option is to divide SpCas9 into two distinct domains, each managed independently (78). 

More compact Cas9 orthologs, including SaCas9, which is about 3.2 kilobase pairs in size, can also be used instead of SpCas9 (110, 120, 121).

Unlike viral vectors, synthetic non-viral delivery systems are less likely to trigger immunological responses and lack the viral machinery needed to incorporate foreign genetic information into the host genome (122). Additionally, it is easy to expand their cargo capacity so that the components of CRISPR/Cas9 can be supplied straight as a ribonucleoprotein (RNP) complex (110, 123, 124) or to combine the donor template, Cas9 nuclease, and sgRNA into a single construct (125, 126). Another advantage of synthetic vectors is their scalability, which makes large-scale manufacturing effective (127, 128). Compared to viral-based techniques, this strategy’s very poor gene delivery efficiency is a significant disadvantage (110, 122, 129). Because of their increased delivery efficiency and reduced potential for unwanted systemic effects, viral vectors remain the favored choice for the bulk of gene therapy clinical studies despite growing interest in synthetic vectors (110, 130, 131).

Significant progress has been made in the clinical translation of CRISPR/Cas9 for CRC, with many delivery systems now undergoing clinical studies. For example, Intellia Therapeutics and Editas Medicine are leading the way in the *in vivo* delivery of CRISPR using lipid nanoparticles (LNPs) and AAV vectors. The viability and safety of systemic CRISPR delivery have been established by Intellia’s NTLA-2001, an LNP-formulated CRISPR treatment for transthyretin amyloidosis (132). Similarly, the promise of viral vectors in precision gene editing is shown by Editas’ EDIT-101, an AAV-delivered CRISPR treatment for Leber congenital amaurosis (133). 

Non-viral delivery of CRISPR/Cas9 components can be achieved by linking the sgRNA-Cas9 complex to specific peptide sequences or encapsulating the genetic material in carriers made from lipids, polymers, or inorganic materials. Delivering CRISPR/Cas9 as a ribonucleoprotein (RNP) complex using synthetic vectors offers a key advantage: it shortens the exposure time to the editing machinery, thereby minimizing the risk of off-target effects (110, 124, 125, 134). Furthermore, by adding surface ligands that identify and bind to specific receptors expressed on target cells, synthetic vectors may be created for targeted delivery to specific cell populations *in vivo* (110, 135, 136). These ligands enable accurate differentiation between healthy and malignant tissues and can be made of chemical compounds, antibodies, aptamers, or proteins/peptides (135, 136).

One recent study illustrating the efficacy of this approach is the coupling of folic acid molecules to polyethylene glycol-succinyl-Chol liposomes, which enabled precise targeting of CRISPR/Cas9 vectors to ovarian cancer cells due to their overexpression of folate receptors (110, 137, 138). Folic acid ligands and folate receptors help cellular absorption by endocytosis and the subsequent intracellular release of the gene-editing components by extending the distance between the vector and the target cell (138, 139). Similarly, transferrin ligands have been added to the surface of liposomal carriers to target ovarian cancer cells that express many transferrin receptors (110, 140, 141).

**Figure 4:**
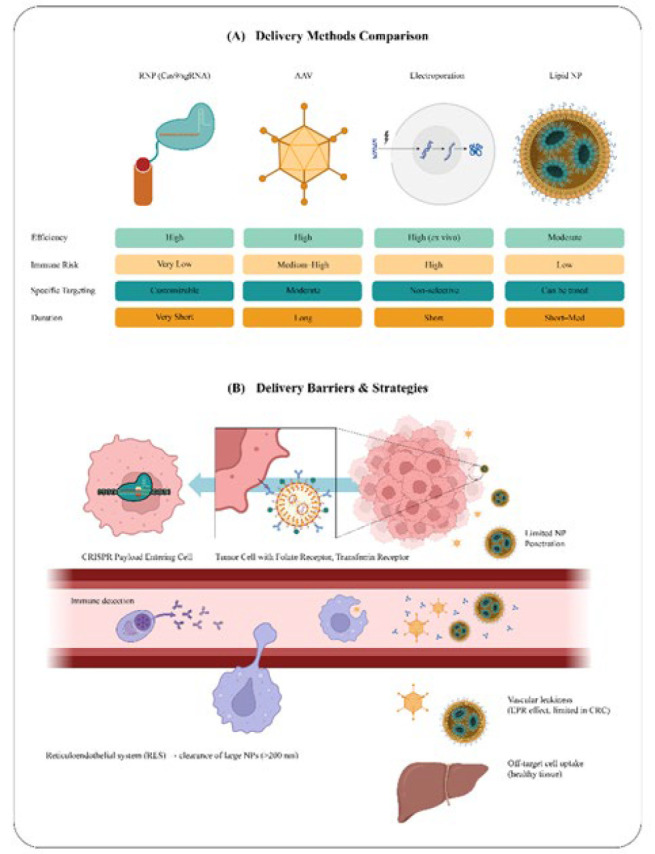
Delivery strategies and barriers for CRISPR/Cas9-based therapeutics in colorectal cancer (CRC)

Furthermore, by allowing them to pass through the blood-brain barrier and alter genes in glioblastoma-associated cells, peptides or antibodies, like Angiopep-2, may be coupled to synthetic vectors to enhance these targeted delivery methods even further (142-144). Additionally, sophisticated cell-based screening methods like systematic evolution of ligands by exponential enrichment (SELEX) have produced new cell-type-specific aptamers, single-stranded DNA or RNA oligonucleotides that function as recognition elements to target osteosarcoma cells specifically *in vivo* (Figure 4A) (110, 145).

Comparisons of current delivery systems need careful consideration. While AAV-DJ has extensive tropism *in vitro* (150), neutralizing antibodies seen in 60% of CRC patients (151) and payload limitations (<4.7 kb) that hinder base editor distribution (152) restrict its practical utility. Although scalable, LNPs have low penetration in the thick stroma of CRC (collagen I: 120-180 μg/mg tissue) (153), a restriction that is not shown in lung cancer models (154). Most importantly, research on the durability of RNP in metastases is contradictory; some studies describe 72-hour activity in PDX models (155), while others demonstrate quick clearance in implants made from cell lines (156). These disparities highlight the necessity for standardized CRC-specific delivery parameters, which most likely result from variations in metastatic biology.

### Benefits and challenges of CRISPR-Cas9 therapy for colon cancer

CRISPR-Cas9 has become a powerful tool for uncovering the specific roles of mutations involved in the development of CRC. It is extensively used to explore the disease’s progression and to map the step-by-step genetic changes that drive tumor formation. This versatile technology enables precise genome editing, allowing researchers to simultaneously add or delete multiple genes. Genome-wide CRISPR-Cas9 knockout screens have shown that, when KRAS is activated, specific gene deletions can either accelerate or suppress tumor growth, revealing key metabolic weaknesses that could be targeted therapeutically. These findings highlight the possibility of using metabolic pathway targeting as a treatment approach in KRAS-mutant CRC (41, 157, 158). The HCT-116 human colon cancer cell line’s β-catenin pathway mutations were corrected using CRISPR-Cas9, which also decreased β-catenin translocation to the nucleus, down-regulated survivin and c-myc production, and restored Wnt phosphorylation. In mouse xenograft models, these genetic alterations dramatically reduced cell proliferation and hindered tumor formation (159).

Four key genes often changed in CRC genes, APC, TP53, KRAS, and SMAD4, have been accurately modified in cultured human intestinal stem cells using CRISPR-Cas9 technology (Table 5). Researchers successfully created tumors with histological characteristics resembling adenocarcinoma by transplanting the altered cells into recipient mice after methodically introducing mutations in these genes using targeted guide RNAs. This technique simplified finding the primary driver mutations leading to tumor formation and progression (41, 160).

Further studies have validated the effectiveness of CRISPR-Cas9 for *in vivo* genome editing and organoid-based transplanting of colon tumors in mice, even in the absence of predisposing genetic abnormalities (158). This gene-editing technique has also helped detect other significant carcinogenic changes, such as mutations in Acvr1b, Acvr2a, and Arid2 (42), as well as disturbances in the MUC5AC-CD44 signaling pathway, which deepens our comprehension of the pathophysiology of CRC (161).

### Challenges of CRISPR-Cas9 therapy for colon cancer

**Challenges and constraints**

Even while CRISPR-Cas9 has great promise for treating colon cancer, several issues still need to be resolved. One of the biggest concerns is the potential for off-target effects, which are inadvertent genetic alterations that may cause genomic instability or even the emergence of additional malignancies. Research on improving CRISPR-Cas9’s specificity and accuracy is still essential to reducing these hazards.

A significant additional difficulty is the efficient delivery of CRISPR components to target cells. Both viral and non-viral delivery methods have drawbacks. Viral vectors, like AAV, effectively transport gene-editing tools but may also trigger immune responses. In contrast, non-viral carriers, such as lipid nanoparticles, are safer but frequently do not reach tumor cells. Resolving these delivery issues is necessary to maximize the therapeutic potential of CRISPR-based treatments (Figure 4B).

*Modifying germline mutations, such as APC*

Editing germline mutations, such as those in the APC gene, which are connected to hereditary CRC syndromes, is one of the significant ethical issues in CRC research using CRISPR technology. Since the patient will be impacted and may be passed on to future generations, editing germline mutations has significant ethical ramifications. By fixing germline abnormalities, gene editing may help prevent hereditary colon cancer. However, there are worries about the long-term effects on the human gene pool and the possibility of unforeseen repercussions. Careful thought must be given to the potential for “designer babies” and the moral implications of altering the DNA in such a way. Furthermore, it is critical to distinguish between improvements that can be seen as a type of genetic alteration for non-medical purposes and therapeutic editing intended to avoid disease (162).

*Consent and the use of patient-derived models (organoids)*

To better understand tumor biology and customize cancer treatments, CRISPR has gained popularity when used in patient-derived models like organoids. However, this also brings up significant moral dilemmas regarding patient consent. Patients must be well aware of the consequences of utilizing their genetic material in organoid development research before donating tumor samples. Particularly when genetic modifications are given to the organoids to research particular medication responses or to mimic treatments, consent has to be explicit and well-informed. The commercialization of these organoid models also raises ethical questions, particularly if patient data is utilized without providing enough recompense or benefit to the patient. Furthermore, maintaining data security and privacy is essential as these models advance, especially when handling sensitive genetic data (163, 164).

*Personalized CRISPR therapy informed consent*

Careful informed consent is necessary for personalized CRISPR-based treatments for CRC, which include editing specific mutations in a patient’s cancer cells, such as KRAS or APC. The treatment’s possible side effects, such as off-target effects (where genes may be changed without the intended purpose) and the potential to change healthy tissues, must be thoroughly explained to patients. The possibility that altered cancer cells can develop in novel ways and give rise to new cancers is another worry (165). When medications are customized to a patient’s genetic profile, informed consent becomes more difficult since patients must be informed of the known and perhaps undiscovered hazards associated with these treatments. Protecting the patient’s autonomy and decision-making authority is essential to ensuring they make well-informed decisions using such experimental medicines (25).

**Bridging the gap to clinical trials**

Three main obstacles stand in the way of the clinical translation of CRISPR/Cas9 for CRC: (I) safety and regulatory concerns, (II) biological barriers unique to each patient, and (III) a lack of clinical trial data that is particular to CRC as opposed to other cancers (166).

*Regulatory and safety hurdles*

Before being used in clinical settings, CRISPR treatments need to address serious safety issues. Studies show that there is a continuing danger of substantial genomic deletions (>100bp) and complex rearrangements at both target and off-target locations, even if tailored variants such as HypaCas9 exhibit enhanced specificity (167, 168). While base editors are more accurate, they can still cause off-target RNA edits across the transcriptome that may interfere with regular biological processes (169).

Viral delivery systems, particularly AAVs, face immunogenicity challenges. Approximately 30-60% of the population shows pre-existing neutralizing antibodies against common AAV serotypes, potentially limiting treatment efficacy. Non-viral alternatives like LNPs exhibit reduced immunogenicity but currently achieve only 10-15% transfection efficiency in solid tumors (63).

*Patient-specific biological challenges*

The molecular heterogeneity of CRC poses particular challenges. Whole-exome sequencing finds more than 200 non-synonymous mutations in each tumor, with significant inter-patient variability seen in KRAS, APC, and TP53 (170). According to single-cell investigations (160), therapeutic resistance can be driven by subclonal populations that comprise only 0.1% of tumor cells. 

Treatment is made more difficult by the CRC microenvironment:

Poor T-cell infiltration is seen in 70-80% of MSS CRC tumors (171). 

Compared to normal tissue, dense collagen matrices decrease nanoparticle penetration by more than 50% (172, 173). 

Anti-Cas9 antibodies are present in up to 58% of patients (174).

*Clinical translation progress*

As of 2024, only a few of the more than 100 ongoing CRISPR clinical studies worldwide are exclusively focused on CRC. Notable instances consist of: 

With objective response rates of 40% in solid tumors, the NY-ESO-1 CAR-T cell study (NCT05309733) has shown promise for adoptive cell treatments in CRC (154). Similarly, in liver cancer studies, LNP-delivered CRISPR treatments have achieved 30% tumor reduction (NCT05210530), indicating possible application to CRC metastases (156). Exa-cel, the first FDA-approved CRISPR treatment for sickle cell disease, has set significant safety standards by reducing vaso-occlusive crises by 94% (67).

Furthermore, ethical, safety, and regulatory issues must be resolved for CRISPR-Cas9 to successfully incorporate into cancer treatment. Before CRISPR-based medicines are authorized for broad clinical use, regulatory agencies enforce stringent preclinical and clinical testing standards to guarantee their efficacy and safety. There are ethical concerns with using gene-editing technology in human medicine, especially when it comes to unforeseen long-term effects.

### Advancements in CRISPR technology

**Base editing**

Base editing is a highly versatile gene-editing technique that enables precise single-nucleotide changes without requiring donor DNA templates or inducing double-strand breaks (DSBs). This makes it especially effective in cells that lack efficient homologous recombination (HDR) repair pathways. Base editors (BEs) are engineered fusion proteins that include a Cas9 nickase, a modified form of Cas9 with an inactivated RuvC domain that ensures targeted and controlled genetic modifications (175-179). A nucleotide deaminase enzyme (175-180) is linked to this mechanism, making it possible to change one nucleotide base into another precisely. During the base-editing process, a guide RNA directs the base editor to a specific genomic DNA sequence. The modified Cas protein then unwinds the target single-stranded DNA, enabling the deaminase to employ deamination to produce site-specific base modifications (175). The two main types of base editors in the first generation were adenine base editors (ABEs) and cytosine base editors (CBEs)(180-184). CBEs comprise a catalytic area derived from cytidine deaminases, like APOBEC1, and a uracil glycosylase inhibitor (UGI) domain. This combination allows for accurate conversion of cytosine (C) to thymine (T), enabling targeted and efficient single-base alterations within the genome (175, 180).

ABEs employ a modified adenine deaminase domain from tRNA-specific adenosine deaminase (TadA), which has been tailored by directed evolution to work on single-stranded DNA (ssDNA) to specifically convert adenine (A) to guanine (G)(175, 184). Base editors minimize the dangers associated with DSBs while providing fewer accidental insertions or deletions (indels), improved precision, and increased efficiency compared to traditional CRISPR-Cas nucleases (167, 175, 185-191).

To increase their activity and lessen the off-target effects brought on by deaminase activity, CBEs and ABEs have undergone several improvements since their original creation (178, 192-199). Advanced base editors may now incorporate cytidine and adenine deaminases simultaneously, expanding the breadth of base editing beyond basic A-to-G or C-to-T conversions (200-202). Furthermore, additional editing techniques have been established, such as base swapping (e.g., C-to-G, A-to-C, T-to-C, and T-to-G); however, the specificity and efficacy of these techniques vary according to the genomic target (175, 203-209).

Swap-type base editing’s effectiveness and specificity vary greatly and mainly depend on the target site’s genomic context (203, 205, 206, 208). BEs have been widely used in thorough genome-wide gene knockout investigations and systematic mutation screening due to their alterations’ predictability, making functional genomic research easier (210, 211). Furthermore, they have gradually been used in clinical research because of their ability to fix harmful single-nucleotide variants or introduce protective genetic modifications accurately. Notably, several preclinical studies have shown the therapeutic potential of base editing, including those carried out in models of non-human primates, highlighting its translational usefulness in precision medicine (212-214). Although base editors provide high precision in genetic modifications, unintended edits can still arise, particularly with the development of more potent editing tools. These unintended alterations are generally categorized based on whether they occur at the intended target site or unintended off-target regions within the genome (215, 216). Furthermore, based on their reliance on Cas9 activity, these impacts may be further categorized, emphasizing the necessity for ongoing improvement to improve editing specificity and reduce undesirable genomic alterations (162, 169, 175, 179, 217-220). Because base editors are used so widely, there is a constant need to improve them, which calls for constant improvements in efficiency, accuracy, and specificity to improve their overall performance (179, 221, 222). Several crucial areas are covered by key research objectives in the development of base editor technology: reducing accidental editing byproducts (180, 192, 193, 196, 215, 223-229), enhancing genomic integrity (221, 222, 230), optimizing editing efficiency (178, 179, 196, 198, 231-234) and fidelity (234, 235) to improve overall accuracy, broadening the spectrum of targetable genomic sites to extend its applicability, increasing the diversity of editable nucleotide substitutions, and refining editing precision to ensure greater specificity in genetic modifications (180, 184, 192, 223, 226-229). Even though many limitations have been significantly lessened by engineering advancements (178,192–199)C or T, Current methods have not yet been able to replace all 12 types of point mutations (209, 236, 237), and they are still not enough to do most conversions, insertions, deletions, and other kinds of genomic modifications (175, 238).

**Prime editing**

Prime editing represents a cutting-edge gene-editing approach capable of making highly precise and versatile DNA modifications. Unlike traditional methods, it can introduce a wide variety of nucleotide substitutions and insert or delete short DNA sequences at specific genomic sites, all without generating DSBs, making it both efficient and less disruptive to the genome (152). Prime editors comprise a protein component and a prime editing guide RNA (pegRNA). One essential part of the prime editing mechanism is pegRNA. It combines an extended RNA template encoding the required change with the targeting power of a regular sgRNA. A primer binding site (PBS) to anneal to the displaced DNA strand, a spacer sequence for DNA binding, and an RNA template for reverse transcription are all components of the pegRNA structure. Without donor DNA templates or double-strand breaks, this architecture allows for accurate alterations (152).

 The protein component consists of a modified Cas9 nickase with the HNH nuclease domain inactivated, linked to an altered reverse transcriptase domain. In addition to providing a programmable RNA template that encodes the desired genetic alteration, pegRNA also guides the editing machinery to the precise genomic region (175). The process begins when the primary editor introduces a single-strand nick at a specific site in the genome, causing a nearby DNA fragment to shift. If the conditions for base pairing are favorable, this displaced DNA strand can bind to the primer binding site on pegRNA. Once hybridized, the DNA fragment acts as a primer for the reverse transcriptase, which extends the sequence using the RNA template embedded within the pegRNA. The newly synthesized DNA strand is then stably incorporated into the genome through natural end-repair and ligation processes (152, 175).

Instead of using deaminases to induce chemical base changes, prime editors (PEs) make targeted genomic modifications through a reverse transcriptase process guided by pegRNA. This process involves three distinct and sequential hybridization steps. First, the prime editor binds to and cleaves the target DNA site, aligning with the pegRNA spacer sequence. Next, the PBS of pegRNA anneals to the 3’ end of the cleaved genomic DNA. Finally, the DNA strand synthesized during reverse transcription hybridizes with the genomic sequence to complete the editing process (175). Compared to base editing and HDR, prime editing’s precision, fidelity, and target specificity are greatly enhanced by this necessity for several base-pairing interactions (31, 239-242). Prime editing’s widespread applicability in genome engineering is demonstrated by its effective application in various model animals and tissue types (175, 243-245).

Rather than relying on deaminases to induce chemical base changes, PEs use a reverse transcriptase mechanism guided by pegRNA to make precise genomic modifications. The prime editing process is characterized by three distinct and sequential hybridization events: first, the prime editor binds to and cleaves the target DNA site in alignment with the pegRNA spacer sequence; second, the PBS of pegRNA anneals to the 3ʹ end of the cleaved genomic DNA; and third, the reverse transcription-synthesized DNA strand hybridizes with the genomic sequence. Recent advancements in understanding the cellular factors that impact prime editing efficiency have resulted in significant progress, contributing to the development of the Prime Editing Guide (246-249)has been improved; adding robust secondary structures at their 3Ϲ terminal to boost structural integrity and lessen deterioration is one such change (250, 251).

**Mitigating off-target effects in CRC therapy**

*High-fidelity cas9 variants*

Designed variations such as eSpCas9 and HypaCas9 efficiently edit oncogenes like KRAS and APC while reducing off-target effects by more than ten times in CRC models (168, 252). For MSI-high CRC subtypes, where genomic instability raises off-target hazards, these variations are especially useful (160).

*Advanced prediction tools*

CRC-specific off-target profiling is now possible thanks to computational techniques like CIRCLE-seq (253) and machine learning platforms, which can identify <5 possible off-target locations for common CRC driver mutations in patient-derived organoids (17).

*Precision delivery systems*

By shortening the period of editing and improving tissue specificity, transient RNP delivery and tumor-targeted nanoparticles reduce off-target effects in CRC. These methods have demonstrated special potential in liver metastasis models of CRC (138, 254).

These tactics, taken together, address the urgent need for safer CRISPR uses in CRC. To close the gap between technological advancements and therapeutic application, future validation should concentrate on clinically relevant CRC models, such as *in vivo* metastatic systems and TP53-mutant organoids (158).

### Utilization of the CRISPR/Cas9 system in CRC therapy

Using the CRISPR/dCas9-VPR plasmid as an expression vector, the fucosyltransferase 4 (Fut4) and Fut9 genes were transcriptionally activated in the MC38 murine CRC cell line (255). When these genes were introduced, Lewis’s antigens were expressed, affecting sialylation and core fucosylation amounts. The HPV16 gene, which is associated with anal cancer, was also expressed in immunodeficient mice to evaluate the CRISPR/Cas9 system’s capacity to stop tumor growth. The delivery of Cas9/sgRNA via AAV vectors, which encoded Cas9, reduced tumor volume by targeting HPV16. These findings suggest that the CRISPR/Cas9 system may be exploited as a therapeutic approach to treat HPV-related human cancers (256).

Although earlier research has shown that AAV-CRISPR may reduce tumors by 80% in HPV16-driven CRC mouse models, some restrictions must be considered before clinical translation can occur (10). First, these immunocompromised models do not replicate important features of actual CRC, including the intricate tumor microenvironment present in microsatellite-stable subtypes (160). Second, pre-existing neutralizing antibodies against AAV in 30–60% of populations (257) and payload capacity restrictions (~4.7 kb) that limit the delivery of sophisticated editing systems provide obstacles for clinical applications (150). On the other hand, recent research employing LNP-encapsulated ribonucleoproteins in patient-derived organoids demonstrated similar effectiveness without immunological toxicity, indicating that delivery optimization has to consider CRC subtype-specific needs.

Using lentiviral CRISPR/Cas9 gene editing in CaCO-2 cells, we targeted Partitioning Defective 3-Like protein (Par3L), a crucial regulator of cell polarity and AMPK signaling that enhances CRC cell survival. To suppress Par3L expression, certain sgRNAs were given via the lentiviral vector (pSpCas9(BB)-2A-GFP)(258). Increased caspase-3 activation indicated that this genetic disruption caused apoptosis and markedly reduced cell growth (*P*<0.01). Additionally, cells lacking Par3L showed increased susceptibility to traditional chemotherapeutic drugs, indicating that Par3L plays a crucial part in treatment resistance (258). Deletion of the Par3L protein caused both cascade-3 expression and cell death. Since Par3L-deficient cells showed increased susceptibility to anticancer chemotherapy, the CRISPR/Cas9 system’s deactivation of these cells via decreasing AMPK signaling may be a potential therapeutic target for cancer treatment. Furthermore, it has been shown that by specifically targeting KRAS mutations, the injection of hyaluronic acid-conjugated CP/Ad-SS-GD/RNP nanocomplexes successfully halted the growth and metastasis of colorectal tumors (Table 6)(254). 

### Prospects and future directions

Several key challenges must be addressed to efficiently deliver CRISPR/Cas9 using various nanoparticle (NP) methods to achieve optimal results. One major hurdle is the complex packaging of the CRISPR/Cas9 system, which is highly anionic, along with issues related to NP size, shape, design, surface characteristics, and stability during circulation. Additional concerns include the overall effectiveness of the gene-editing delivery system, its potential immunogenicity, and possible *in vivo* toxicity when different types of NPs are used. Another significant obstacle is the body’s rapid clearance systems, which quickly detect and remove NPs from the bloodstream before they can reach their target cells (166, 261).

The effectiveness of cellular uptake of CRISPR/Cas9-loaded NPs is influenced by various factors, such as vascular flow, diffusion patterns, adhesion properties, and velocity distribution, all of which are significantly affected by the cargo size. Nanoparticles larger than 200 nm are typically cleared by the body’s RES, accumulating in the liver and spleen. Therefore, an ideal CRISPR/Cas9 nanoparticle formulation should possess key characteristics, including prolonged circulation time in the bloodstream, efficient penetration into tumor tissue, high cellular uptake, and successful endosomal escape. This ensures that the CRISPR/Cas9 system can be released into the cytoplasm, optimizing its therapeutic effectiveness (262, 263). 

Despite the widely recognized potential of CRISPR/Cas9 genome editing technology, several doubts about its efficacy and safety necessitate more investigation. Off-target effects, in particular, are a major barrier to the clinical application of the CRISPR/Cas9 system and its further translation into therapeutic contexts (264). The Cas9 protein frequently causes cleavage at off-target regions because the guide RNA is associated with undesirable chromosomal loci and has a somewhat higher tolerance for sequence mismatches (265). One kind of genotoxicity is off-target changes brought on by CRISPR/Cas9 genome editing. Atypical chromosomal rearrangements and unexpected large-scale deletions may occur in cells modified using the CRISPR/Cas9 system (264). Both human and mouse cell lines have shown significant deletions and intricate genomic rearrangements, including insertions and inversions, in regions near and distant from the target cleavage sites (167).

To increase the specificity of CRISPR/Cas9, efforts are frequently made to develop more complex Cas9 nuclease variants with better guide RNA designs that can recognize a wider variety of PAM sequences. These developments are coupled with enhanced delivery methods that are intended to more effectively target certain cell types (5). It is believed that the recently created HypaCas9 and xCas9 variants have improved targeting efficiency and precision genome-editing capabilities (168, 266). Furthermore, it has been shown that new CRISPR/Cas9 inhibitors are required to control genome editing efficiently, and more advantageous substances are expected to be found in the future (266). Furthermore, sophisticated methods like BLESS, Digenome-seq, GUIDE-seq, and HTGTS have been developed to more precisely forecast possible off-target areas and evaluate gene editing results (166, 267).

Significant questions remain in the field of genetic engineering regarding the effectiveness and clinical applicability of genome editing with CRISPR/Cas9. A significant limitation of this technique is its reliance on a specific PAM, particularly the NGG sequence, which is essential for accurate target site recognition and cleavage. The relatively small number of PAM sequences that CRISPR/Cas9 can recognize has historically constrained its use. However, the development of engineered variants, such as xCas9, has significantly expanded the range and flexibility of this genome-editing platform by enabling recognition of a broader array of PAM sequences, including GAA, GAT, and NG (166). It is anticipated that future developments in CRISPR/Cas9-based techniques will be crucial in identifying new oncogenic biomarkers and unidentified genes linked to cancer, which will make it easier to create customized treatment plans (166). This genome-editing technology holds significant potential for identifying new drug targets and understanding their molecular interactions, which could lead to the development of innovative cancer therapies. Moreover, CRISPR/Cas9’s ability to precisely modify noncoding regions of the genome is expected to enhance our understanding of regulatory elements and their roles in carcinogenesis. A comprehensive analysis of the biological changes induced by CRISPR/Cas9 will provide valuable insights into the genetic and epigenetic mechanisms that drive cancer development and metastasis, particularly through the targeted creation of driver mutations and pathogenic variants. Continued progress in CRISPR/Cas9 delivery technologies will further improve its clinical applicability, enabling its integration into treatments for various diseases, including cancer (166, 268).

### Challenges and limitations of crisper in CRC

The CRISPR-Cas (Clustered Regularly Interspaced Short Palindromic Repeats-CRISPR-associated protein) technology has emerged as a revolutionary genome-editing tool with significant potential in cancer research and therapeutics, including CRC. Despite its promising applications ranging from functional genomics to gene therapy, several challenges and limitations still hinder its clinical translation in CRC treatment. Moreover, integrating artificial intelligence (AI) is becoming increasingly pivotal in addressing these challenges and optimizing CRISPR-based interventions (269).

***Off-target effects and genetic mosaicism***

One of the foremost concerns in CRISPR applications is the occurrence of **off-target effects**, where unintended genomic sites are edited due to sequence homology or errors in gRNA design. In the context of CRC, such inaccuracies may disrupt tumor suppressor genes or activate proto-oncogenes, exacerbating the disease or introducing additional oncogenic mutations (270). This is especially critical when editing somatic cells *in vivo*, where the specificity of gene targeting determines safety and efficacy. Furthermore, **genetic mosaicism,** the presence of a mixture of edited and unedited cells, complicates therapeutic outcomes. Mosaicism can arise from incomplete editing or the use of CRISPR in post-zygotic stages, limiting the uniformity and reproducibility of gene correction strategies in CRC models.

***Delivery system limitations***

Efficient delivery of CRISPR components into colorectal tumors remains a major hurdle. Viral vectors (e.g., AAV, lentivirus) offer high transduction efficiency but carry immunogenicity risks, insertional mutagenesis, and size limitations. Non-viral vectors such as lipid nanoparticles and polymer-based carriers present lower immunogenicity but often suffer poor delivery efficiency in solid tumors like CRC (271). Furthermore, the TME in CRC, characterized by hypoxia, fibrosis, and immune suppression, can impair the uptake and distribution of CRISPR reagents (272, 273).

***Tumor heterogeneity and evolution***

CRC exhibits substantial **intertumoral and intratumoral heterogeneity**, resulting in variable CRISPR editing outcomes. Diverse mutational profiles among CRC subtypes (e.g., microsatellite instability-high (MSI-H) vs chromosomal instability (CIN) tumors) may demand highly personalized gRNA designs (274). Moreover, as tumors evolve during progression and treatment, the targeted mutations may become obsolete, reducing the therapeutic relevance of initial edits. Additionally, clonal selection following CRISPR-induced DNA damage may inadvertently select for more aggressive or treatment-resistant subpopulations, complicating long-term control of CRC.

***Ethical, regulatory, and safety concerns***

The ethical implications of germline editing and the potential misuse of CRISPR technology in CRC are subjects of ongoing debate. Regulatory bodies emphasize stringent preclinical validation and monitoring of off-target effects, long-term safety, and immune responses. Moreover, patients with CRC may harbor underlying genetic instability (e.g., Lynch syndrome), necessitating careful evaluation of the broader genomic impacts of CRISPR edits (275). The lack of universally accepted standards for CRISPR clinical trials, especially in oncology, hinders its implementation. Risk-to-benefit ratios, patient selection criteria, and endpoints must be clearly defined to facilitate regulatory approval and public trust.

***Limitations in model systems and experimental reproducibility***

Much of the current CRISPR research in CRC relies on ***in vitro* cell lines and mouse models**, which may not fully recapitulate human tumor complexity. Differences in immune systems, TME, and genetic background between models and patients can lead to discrepancies in therapeutic response and editing efficiency. Reproducibility remains a concern, as CRISPR outcomes can vary depending on gRNA sequence, delivery method, and experimental conditions (276).

**Transformative role of artificial intelligence in overcoming CRISPR limitations**

Amid these challenges, **artificial intelligence (AI)** is emerging as a transformative force in enhancing the accuracy, efficiency, and safety of CRISPR applications in CRC. AI-powered tools can process vast genomic data to optimize gRNA design, predict off-target effects, and model genome editing outcomes with unprecedented precision (277, 278).

### Improved guide RNA design and off-target prediction

AI algorithms, particularly those based on machine learning and deep learning, can analyze large datasets to identify optimal gRNA sequences with high on-target activity and minimal off-target risk. Tools like DeepCRISPR, CRISPR-Net, and Elevation use neural networks trained on empirical data to predict editing outcomes and reduce experimental trial-and-error. This is crucial for CRC, where precision is essential to avoid exacerbating malignant transformation (279).

### Integration with multi-omics and big data

AI facilitates the integration of multi-omics data, including genomics, transcriptomics, and epigenomics, to uncover actionable targets and pathway interactions in CRC. By leveraging AI for biomarker discovery, researchers can prioritize editing targets with the highest therapeutic potential and minimal systemic impact (280).

### Personalized CRISPR therapeutics

AI enables personalized CRC therapy by mapping patient-specific mutations and modeling the impact of CRISPR edits *in silico*. Algorithms can simulate therapeutic interventions and predict resistance mechanisms, allowing for preemptive modifications to treatment strategies. This personalization is particularly vital in CRC, which exhibits variable genetic and epigenetic landscapes across patients.

### Optimizing delivery systems

Machine learning can also assist in engineering more effective and tumor-specific delivery vehicles. By analyzing physicochemical properties, cellular uptake data, and biodistribution patterns, AI can guide the development of nanoparticles or viral vectors tailored for CRC tissues.

### Enhancing preclinical and clinical trial design

AI-driven platforms can enhance the design of CRISPR-based clinical trials by stratifying patients, predicting outcomes, and identifying potential safety issues before trial initiation. Virtual simulations using AI models reduce the need for extensive animal studies and improve the translation of findings to human CRC.

## Conclusion

The advent of CRISPR/Cas9 genome-editing technology has revolutionized CRC research and therapy by enabling previously unheard-of precision in genetic modifications to restrict tumor development, increase medicine responsiveness, and identify novel oncogenic mechanisms. The technology’s potential for targeted gene disruptions and pathway alterations that hasten cancer development is demonstrated by the successful application of CRISPR/Cas9 in CRC models, such as MC38 murine and CaCO-2 human CRC cell lines. Despite its revolutionary potential, CRISPR/Cas9’s clinical translation is still hampered by significant issues such as immunogenicity, off-target consequences, and delivery constraints. To maximize its therapeutic efficacy, these obstacles must be overcome by creating exact CRISPR variants, improved guide RNA designs, and novel nanoparticle-based delivery methods. Furthermore, CRISPR/Cas9 has excellent potential for discovering new CRC biomarkers and regulatory components, opening the door for precision oncology approaches that customize therapies for each patient’s unique characteristics. CRISPR/Cas9 has the potential to completely change the field of CRC treatments as research into its safety and applicability advances. It is a potent tool for both basic studies of cancer biology and the creation of next-generation targeted treatments. Future developments in delivery routes and genome-editing precision will be crucial to convert CRISPR/Cas9 from a promising experimental tool into a clinically feasible therapeutic strategy for CRC.

This figure illustrates the three key steps of the CRISPR/Cas9 gene-editing process: (9) Target Sequence Identification: Cas9 is guided to the target DNA sequence next to the PAM site by the sgRNA (25). Cas9 causes a DSB at the target site in a process known as precise endonucleolytic cleavage (26). The repair processes Homology-Directed Repair (HDR) and Non-Homologous End Joining (NHEJ), which result in genetic alterations such as insertions, deletions, or mutations, are highlighted in subsequent DNA repair via cellular mechanisms (9, 25)

**Table 1:** Comparison and difference of CRISPR editing techniques

Feature	CRISPR/Cas9	Base editing	Prime editing	Reference
Mechanism	Creates double-strand breaks	Direct nucleotide conversion	Precise small edits without DSBs	(27)
Editing efficiency	Moderate	High	High	
Off-target effects	High	Low	Very low	
Applications	Gene knockout, large edits	Single-nucleotide corrections	Small insertions/deletions	

**Table 2:** Applications of CRISPR technology in CRC research and therapy

Category	Target/Application	Mechanism	Outcome/Impact	Clinical potential	Reference
Target identification	Functional genomics screens for CRC-related genes	CRISPR knockout or activation libraries identify genes essential for tumor growth or drug resistance	Discovery of novel therapeutic targets (e.g., oncogenes, tumor suppressors)	Guides the development of targeted therapies and biomarkers	(22, 27)
Oncogene inactivation	Oncogenes (e.g., KRAS)	CRISPR knocks out or edits oncogenes to inhibit tumor growth	Reduced proliferation, invasion, and metastasis of CRC cells	Potential to target "undruggable" oncogenes like KRAS	(42)
Tumor suppressor reactivation	Tumor suppressor genes (e.g., TP53, APC)	CRISPR corrects mutations or restores the function of tumor suppressors	Enhanced apoptosis and reduced tumorigenicity	Restores normal cellular regulation in CRC	(43, 44)
Immunotherapy enhancement	Immune checkpoint molecules (e.g., PD-1, CTLA-4)	CRISPR knocks out immune checkpoint genes in T cells to enhance anti-tumor activity	Improved T-cell-mediated killing of CRC cells	Boosts efficacy of adoptive cell therapies (e.g., CAR-T cells)	(41, 45, 46)
Drug resistance reversal	Drug resistance genes (e.g., EGFR, ABC transporters)	CRISPR disrupts genes conferring resistance to chemotherapy or targeted therapies	Sensitizes CRC cells to existing treatments	Improves response rates to standard therapies	(47)
Microenvironment modulation	Stromal cells, cytokines, and hypoxia-related genes	CRISPR edits genes in the tumor microenvironment (TME) to reduce immunosuppression	Enhanced T-cell infiltration and reduced tumor growth	Complements immunotherapy and reduces CRC recurrence	(48)
Precision medicine	Patient-specific mutations (e.g., APC, KRAS)	CRISPR corrects or edits patient-specific mutations in CRC cells	Personalized therapy tailored to individual genetic profiles	Paves the way for individualized CRC treatments	(49)

This schematic representation summarizes the spectrum of gene targets, editing approaches, and functional outcomes in CRISPR/Cas9 applications for CRC. Gene targets are categorized by therapeutic goal: oncogene inactivation (KRAS, MYC, BRAF, WHSC1), tumor suppressor reactivation (TP53, APC, TFAP2A), drug resistance reversal (EGFR, ABCG2, PUM1, DDX5), immune modulation (PD-1, CTLA-4, PHF8), and microenvironmental modification (Fut4, Fut9, TGF-β). Editing approaches include gene knockout using Cas9/sgRNA, transcriptional activation via dCas9-VPR, base and prime editing for precise corrections, and CRISPR screening for functional genomic analysis. Functional outcomes include reduced tumor cell proliferation, enhanced apoptosis, blocked metastasis, and restored drug sensitivity, highlighting CRISPR/Cas9’s role in precision oncology (26, 27, 31, 41)

**Table 3:** Delivery strategies for CRISPR/Cas9 in colorectal cancer (CRC) therapy

Delivery Method	Advantages	Limitations	CRC-Specific Challenges	Mitigation Strategies	Clinical Research Status (CRC)	Reference
AAV	High efficiency, long-term expression	pre-existing immunity (30-60% of CRC patients), and limited cargo capacity (<4.7 kb)	Liver tropism restricts the ability to target tumors, and neutralizing antibodies (NAbs) decrease effectiveness	1. Capsid engineering to avoid NAbs (AAV-DJ, AAV-LK03) 2. IdeS enzyme or plasmapheresis to remove NAbs 3. Promoters unique to tumors (such as CEA-driven)	Preclinical for CRC (CRC) (e.g., HPV16-driven tumor suppression); phase I for genetic diseases (NCT04601051)	(10, 65-68)
Lipid nanoparticles (LNPs)	Non-viral, safe, scalable	Low transfection efficiency (~10–15%) in solid tumors	Permeation is hindered by dense CRC stroma (collagen I: 120–180 μg/mg tissue)	1. Conjugation of hyaluronic acids (e.g., CP/Ad-SS-GD/RNP) 2. Collagenase and other ECM-modifying enzymes 3. PEGylation to improve blood flow	KRAS-targeted LNPs in PDX models are an example of preclinical, phase I/II for genetic illnesses (NCT05232955)	(69, 70)
Electroporation	Direct genome targeting	High cytotoxicity, invasive	restricted to tumors that are easily accessible (e.g., surface metastases)	1. Optimized pulse parameters 2. Combination with immunomodulators (e.g., anti-PD-1)	Trials of *ex vivo* T-cell editing (NCT05309733); not yet for *in vivo* CRC	(71, 72)
Polymeric nanoparticles	Improved stability, tunable properties	restricted tumor selectivity and possible accumulation in the liver	In hypoxic CRC locations, poor penetration	1. Ligand conjugation (transferrin, folate, etc.) 2. Polymers that react to pH for TME targeting 3. Delivery in conjunction with stroma-modulators	No CRC-specific studies have yet been conducted; preclinical (e.g., PLGA-CRISPR in organoids)	(73)
CRISPR RNPs (Ribonucleoproteins)	Immediate gene editing, minimal off-targets	Minimal off-targets, transient activity	Quick removal of liver metastases	1. RNPs targeted by peptides or antibodies (such as anti-EGFR) 2. Stabilizing supramolecular polymers	NCT05210530, the first-in-human study; preclinical success in liver metastases of CRC	(74, 75)

(A) Naïve T Cell Priming in CRC: T-cell priming is the process where immune cells called T-cells are activated by APCs (antigen-presenting cells), which present cancer-specific signals, in this case, those linked to CRC, to the T-cells. CRC tumors often express specific tumor antigens, such as carcinoembryonic antigen (CEA), which T-cells recognize. The lymph nodes near CRC tumors are the sites where T-cells first encounter these antigens. The key point is that the immune system’s initial T-cell response to CRC-specific antigens is often hindered by the tumor’s ability to escape immune detection. CRISPR technology can potentially enhance this initial T-cell activation by modifying T-cells ex vivo to make them more responsive to CRC tumor antigens.

(B) *Ex Vivo* CRISPR Editing of T Cells: This part explains how CRISPR/Cas9 can be used to genetically modify T-cells outside the body (ex vivo) before reintroducing them to target the tumor. The focus is on immune checkpoint molecules like PD-1 and CTLA-4, which are frequently up-regulated in CRC tumors to prevent the immune system from attacking the cancer cells. CRC tumors often express PD-L1, an immune checkpoint protein that binds to PD-1 on T-cells and inhibits T-cell activity. In CRC, this results in “immune escape,” where the tumor evades immune destruction. By editing the T-cells to knockout PD-1 or CTLA-4 using CRISPR, we can enhance T-cell cytotoxicity and restore the immune system’s ability to fight cancer. CRISPR gene editing allows for the knockout of these inhibitory molecules (like PD-1) on T-cells, thereby overcoming the tumor’s mechanisms of immune suppression. CRISPR can up-regulate beneficial cytokines (e.g., IL-12) or down-regulate immunosuppressive factors (e.g., TGF-β), often abundant in CRC’s tumor microenvironment

C) Within the Tumor Microenvironment: This section emphasizes how CRISPR-edited T-cells behave once they infiltrate the tumor microenvironment. The tumor microenvironment in CRC is often immunosuppressive, with factors like TGF-β and the expression of PD-L1 that suppress the immune response

**Table 4:** CRISPR-based approaches in enhancing colorectal cancer (CRC) immunotherapy

Strategy	Target	Mechanism	Expected outcome	Reference
Immune checkpoint editing	PD-1	Knockout of inhibitory checkpoints	Enhanced T-cell response	(106)
Cytokine modulation	IL-2, IL-12, TGF-β	CRISPR-induced overexpression/suppression	Improved immune activation	(43, 107, 108)
CAR-T cell engineering	KRAS, HER2	Enhances tumor-targeting specificity	Increased tumor cytotoxicity	(109)

Panel (A) compares four major delivery methods for CRISPR/Cas9 systems: ribonucleoprotein complexes (RNPs), adeno-associated viruses (AAV), electroporation, and lipid nanoparticles (LNPs). Each method is assessed for efficiency, immune risk, targeting specificity, and the duration of gene expression. RNPs offer high editing precision with minimal immunogenicity and transient activity, making them suitable for ex vivo or short-term in vivo applications. AAVs provide efficient and long-lasting delivery but are limited by immune recognition and packaging capacity (~4.7 kb). Electroporation achieves high ex vivo delivery rates but lacks targeting specificity and can cause cell damage. LNPs present a tunable and low-immunogenic platform for in vivo applications, although they tend to have only moderate efficiency. Panel (B) illustrates the biological and physiological obstacles to the efficient delivery of CRISPR/Cas9 in vivo for CRC. These include the reticuloendothelial system (RES) for immune detection and clearance, the compact extracellular matrix of colorectal tumors that limits tumor penetration, off-target accumulation in non-tumor tissues like the liver, and the relatively low vascular permeability in CRC, which limits the extravasation of nanoparticles. Potential strategies to overcome these barriers include using ligand-functionalized nanoparticles targeting specific receptors on tumor cells (e.g., folate or transferrin receptors) and adopting compact or split-Cas9 systems to circumvent delivery size constraints (146-149)

**Table 5:** Applications of CRISPR/Cas9 in colorectal cancer research and therapy

Application	Target Genes/Pathways	Mechanism	Outcome/Impact	Reference
Gene knockout	KRAS, TP53, APC	Disrupts oncogenes/tumor suppressors	Reduced tumor growth	(71)
Gene correction	APC, TP53	Restores the function of tumor suppressors	Enhanced apoptosis and genomic stability	(27)
Immunotherapy enhancement	CTLA-4	Enhances T-cell response	Improved tumor clearance	(41)
Overcoming drug resistance	EGFR, ABC transporters	Sensitizes CRC cells to therapy	Increased drug efficacy	(47)
Tumor microenvironment modulation	Cytokines, TGF-β	Alter immune interactions	Enhanced immune infiltration	(43)

**Table 6:** Critical comparison of preclinical vs clinical CRISPR/Cas9 studies in colorectal cancer (CRC)

Study Type	Editing target	Delivery method	Key findings	Clinical translatability	Limitations	Reference
Preclinical	KRAS (G12D)	AAV-DJ	80% tumor suppression in CRC models induced by HV16	Restricted by payload limitations (<4.7 kb) and pre-existing AAV immunity (30-60% of patients)	Immunogenicity hazards; lacks human TME complexity	(217)
Preclinical	TP53/APC	Lentivirus (organoids)	Adenocarcinoma recapitulated in xenografts	High significance for models derived from patients but difficulties with scalability for clinical application	Low effectiveness of *in vivo* administration; safety issues with viral integration	(160)
Preclinical	PD-1/CTLA-4	Electroporation (CAR-T)	Increased cytotoxicity of T cells in murine models	Adoptive cell treatment shows promise; however, solid tumors (such as CRC) need to be optimized	In MSS-CRC, poor T-cell infiltration, immunosuppressive TME	(259)
Preclinical	Par3L (AMPK pathway)	LNP-RNP	The chemosensory effect of CaCO-2 cells	FDA-approved LNPs, such as Onpattro®, have limited penetration due to the collagen-rich CRC stroma	Low editing effectiveness in tumor cores with hypoxia	(258, 260)
Clinical	NY-ESO-1 (CAR-T)	Electroporation in *ex vivo*	40% response in solid tumors	directly relevant to CMS4 subtype (immune-hot) CRC	restricted to individuals who match HLA; expensive production	(NCT05309733)
Clinical	KRAS (LNP-CRISPR)	Lipid nanoparticles	30% decrease in liver metastases from tumors	Promoting systemic administration to target metastatic CRC	requires frequent dosage; short-term effects	(NCT05210530)

## Declaration

We have not used any AI tools or technologies to prepare this manuscript.
